# Establishment of the first speed breeding facility in Central Asia: development of an accessible bread wheat (*Triticum aestivum* L.) protocol for resource-limited research institutions

**DOI:** 10.3389/fpls.2026.1876304

**Published:** 2026-07-17

**Authors:** Kanat Yermekbayev, Aibek Abduakassov, Madi Shoken, Zhuldyz Sartbayeva, Kuat Baimyrzayev, Makpal Bauyrzhan, Saule Abugalieva, Yerlan Turuspekov, Zahid Mahmood

**Affiliations:** 1 Zhetysu University named after I.Zhansugurov, Institute of Biotechnology and Ecology, Taldykorgan, Kazakhstan; 2 Laboratory of Molecular Genetics, Institute of Plant Biology and Biotechnology, Almaty, Kazakhstan; 3 Crop Sciences Institute, National Agricultural Research Centre, Islamabad, Pakistan

**Keywords:** Central Asia, light intensity, light spectrum, nutrient management, potting substrate, protocol, speed breeding, wheat (*Triticum aestivum* L.)

## Abstract

**Introduction:**

Speed Breeding (SB) has become an essential tool for rapid breeding pipeline. However, the practical implementation of existing wheat (*Triticum aestivum* L.) SB protocols is largely constrained by a dependence on specialized plant care facilities. This study provides detailed instructions for growing wheat under SB conditions that can be implemented by institutions lacking dedicated technical support departments.

**Methods:**

The first Central Asian SB facility was established at Zhetysu University named after Iliyas Zhansugurov. For the identification of an effective plant growing substrate, sixteen treatments were developed. The nutrient management of the most suitable potting mix included two steps: i) identification of the importance of Nitrogen-Phosphorus-Potassium (NPK) supplementation, and ii) identification of optimal timing and nutrient dosage. Fertilizers were supplied at 4 different time intervals. Plants were supplemented with a foliar spray of calcium nitrate and copper and were hand-watered once a day. The plant material included seven wheat genotypes. Developmental dynamics and grain yield were evaluated under two, 1.5-and 2.0-meter, light-to-bench distances. A germination test was conducted at different time points after flowering.

**Results:**

In this study, we report the establishment of the first SB facility in Central Asia. The developed cost-effective potting mix contained 65% FFPM, 18% perlite, 12% sand, and 7% bio humus per L^-1^. An additional NPK supplementation under the 21d_L time interval significantly increased effective tiller number (ETN, p=1x10^-5^), number of kernels per plant (NKP, p=1x10^-8^) and grain weight per plant (GWP, p=2x10^-5^) compared to non-fertilized control (NF). The ETN of plants grown under the 1.5 m treatment was significantly higher (p=3x10^-4^) than that of in 2.0 m condition. This resulted in an 83.3% increase in ETN (p=2x10^-4^), 145% in NKP (p=8x10^-3^) and 127% in GWP (p=6x10^-3^) without compromising thousand grain weight (TGW, p=8x10^-1^), highlighting a potential positive effect of proximity to a 1.5 m light-bench under SB conditions. The light intensities in the range of 500–700, depending on the developmental stage, supported healthy wheat growth under SB conditions.

**Discussion:**

This study findings support sustainable regional and global wheat breeding programs aiming to incorporate the speed breeding technique into their breeding schemes, specifically in underrepresented regions.

## 1 Introduction

As of 2025, the global population is estimated at 8.4 billion and is anticipated to reach 9.9 billion by 2050 (United Nations). Consequently, the demand for food is projected to increase substantially. Out of approximately 300000 known edible plant species worldwide, a small subset, around 200, is consumed for human nutrition. Among them, rice, maize, and wheat collectively supply 60% of the energy consumed globally (Voss-Fels et al., 2019). Therefore, stagnant or declining grain yields in these crop species are detrimental to the global food value chain, especially for low-income countries facing rising population pressure (Ray et al., 2013). Population-driven pressures, combined with adverse climate change impacts, including droughts, floods, and extreme temperatures, pose a significant threat to global food security (Shanmugavel et al., 2022). Hence, plant breeding progress should outpace, or at least keep pace with, these rapidly emerging challenges.

To address the need for rapid crop improvement, Speed Breeding (SB) – an accelerated, controlled-environment technique – has emerged as a promising tool. SB was designed to shorten crossing and inbreeding significantly compared to traditional breeding methods, by which the development of improved crop varieties usually requires over a decade (Ghosh et al., 2018; Watson et al., 2018). The SB technique made it possible to obtain up to 6 generations of many crop species per year, compared to only 1 to 2 generations with traditional breeding methods (Watson et al., 2018). SB can be integrated with modern genomic studies (gene editing, genotyping, genomic prediction, and so on) to accelerate the genetic gain per unit time (Potts et al., 2023). The accelerated growth under SB is primarily induced by i) extended photoperiod with optimal light spectra and intensity through powerful LED lighting systems, ii) controlled temperature regimes, and iii) a high-quality potting mixture. The further reduction of the growing duration could be achieved through the early harvest of immature seeds (Ghosh et al., 2018; Watson et al., 2018; Gopinathan et al., 2025).

Extended photoperiod using LED grow lights is a key SB component for rapid generation turnover. Its quality and spectral distribution significantly influence plant growth and development, affecting specific morphological traits such as internode length, leaf thickness, chlorophyll content, floral induction, and flower development in photoperiodic plants (Fowler et al., 2001; Lagercrantz, 2009). Different crop species, as well as different genotypes within each species, have varying photoperiod requirements for flowering and reproductive success (Webb et al., 2019). A change in photoperiod received by photoreceptors in many plants can trigger early development of their reproductive systems (Fowler et al., 2001). This helps to maximize physiological development by aligning plant growth with seasonal environmental changes. An extended light (22 hours of daylight and 2 hours of dark) arrangement under SB was chosen to support the functional expression of all circadian clock genes required for normal plant development (Ghosh et al., 2018). The circadian clocks, in turn, are vital for following photoperiod and react quickly to environmental changes (Lagercrantz, 2009; Webb et al., 2019).

Besides the photoperiod, temperature is an essential component of SB that influences germination rate, plant growth, the transition from vegetative to reproductive phases, successful pollination, and final grain yield (Pipattanawong et al., 2009). Generally, an increase in temperature promotes vegetative and reproductive growth in many plant species (Hatfield et al., 2011). However, the precise control of temperature regimes under SB is essential (Ghosh et al., 2018). For balanced development and accelerated plant growth, a temperature range of 18–22 °C is approximately optimal in current SB protocols for wheat (Watson et al., 2018). High temperatures (22 °C) are used to accelerate vegetative growth, and low temperatures (18 °C) are maintained during reproductive development (Hickey et al., 2019). Therefore, during the 2-hour dark period, it is vital to maintain a lower temperature to improve overall plant health (Ghosh et al., 2018; Watson et al., 2018).

A high-quality potting mixture should offer the perfect blend of nutrients and texture for healthy plant growth in SB (Bayhan et al., 2025). Balanced texture of potting substrate enhances aeration, water infiltration, and proper retention, allowing rapid root development, thus overall plant health (Gohardoust et al., 2020). Therefore, a good potting substrate prevents soil-related issues like oxygen deficiency, limited leaching, mold, and algal growth (Verhagen, 2013; Bayabil et al., 2021). Availability of sufficient nitrogen (nitrate or ammonium), phosphorus, and potassium alongside micronutrients in the potting mixture has a great impact on the transition from vegetative to reproductive stage, flowering time regulation, and biomass accumulation (Zhang et al., 2023; Baek et al., 2026). Therefore, properly proportioned application of nutrients during plant growth significantly expedites rapid generation advancement and improves grain yield under SB (Kigoni et al., 2023; Chaudhary and Sandhu, 2024).

Among cereal crops, wheat holds a significant importance for sustaining the global food security. Kazakhstan is one of the world’s major wheat producers supplying high quality grain. Thus, wheat plays a crucial role in the country’s agricultural sector and economy, underpinning food security, rural livelihoods and export revenues (Kazakhstan: Agricultural Policy Monitoring and Evaluation 2025 | OECD). Moreover, it is a staple food for Kazakhstan’s population, forming the basis of traditional diets, including bread and pasta products (Bonjean and Angus, 2001; Morgounov et al., 2001). Wheat as a dominant exporting crop, contributes significantly to Kazakhstan’s GDP and rural employment. In 2025, Kazakhstan’s agricultural sector contributed approximately 4 – 6% of GDP, with wheat being the largest component (Bureau of National Statistics or Ministry of Agriculture, 2025, Kazakhstan). Despite this significance, the wheat-growing area has gradually shrunk from 14 to 12 million hectares due to reduced profitability. Almost 40% of the officially registered spring wheat varieties originate from foreign breeding programs (Savin et al., 2025). In addition, the currently grown commercial cultivars may not satisfy producers’ quality requirements – the main determinant of product value – or may simply lack the genetic resilience needed to cope with changing climatic conditions (Savin et al., 2025, 2026). Therefore, there is an urgent need to develop new wheat varieties with improved quality and grain yield using accelerated breeding approaches such as speed breeding.

Although the SB method has been available in plant breeding since 2013, the sophisticated SB protocol was published in 2018 (O’Connor et al., 2013; Ghosh et al., 2018; Watson et al., 2018). Since then, the SB protocol has been tailored for many plant species, including bread wheat, durum wheat, barley, soybean, canola, rice, cotton, and pigeon pea (Watson et al., 2018; Kabade et al., 2024; Bayhan et al., 2025; Wang et al., 2025; Sharma et al., 2026). Notably, these protocols were based on specific gene pools adapted to certain geographies and thus may require to be fine-tuned when applied to other germplasm panels due to variations in population structure. Moreover, existing SB protocols for wheat may be difficult for plant researchers to implement, especially in research institutions lacking specialized plant care and other support departments (Potts et al., 2023). Therefore, the objective of this study was to discuss the establishment of the first SB facility in Central Asia, and summarize the challenges and notable achievements in adapting the SB system and protocol to wheat genotypes bred for a wide range of wheat-growing environments. Consequently, we highlighted the optimum light-to-bench distance and demonstrated the importance of adjusting light spectrum and intensity during early stages of wheat development under SB conditions. Moreover, we developed a cost-effective potting substrate and improved its nutrient composition. Results revealed that these SB components collectively play a crucial role in successful plant growth under SB. In addition, our improved in-house SB protocol, initially fine-tuned on Kazakhstani, Pakistani, and UK wheat varieties, was confirmed effective across a diverse wheat germplasm panel representing Uzbekistan, Kyrgyzstan, Turkmenistan, Afghanistan, Tajikistan, Russia, Turkiye, Ukraine, and China. This highlights opportunities to extend the knowledge gained from this work to other developing nations, thereby strengthening their local pre-breeding and breeding processes by adopting the SB technique.

## 2 Materials and methods

### 2.1 Experimental location and design of the speed breeding facility

**Figure 1:**
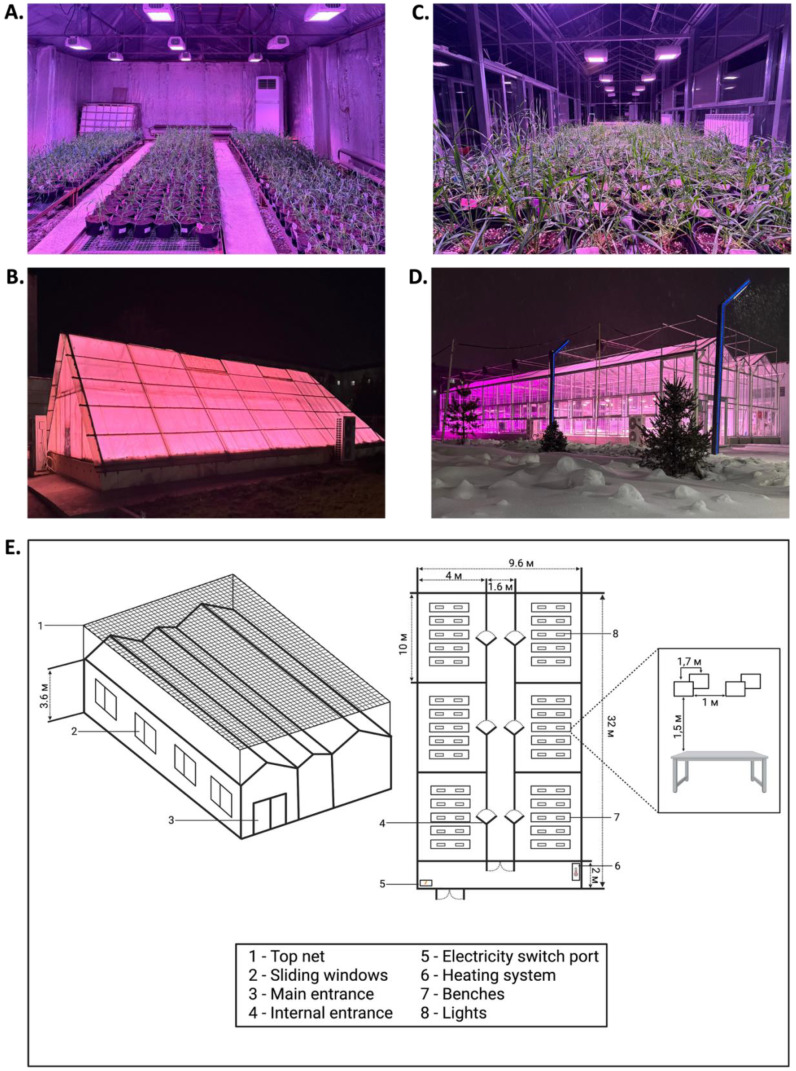
Outlook of ISBF and GSBF at ZU. **(A, B)** are outer and inner view of ISBF respectively. **(C, D)** are outer and inner view of GSBF respectively. **(E)** 2D and 3D view of GSBF.

All experiments were carried out in an indoor and greenhouse Speed Breeding Facilities at Zhetysu University named after Iliyas Zhansugurov (ZU), Taldykorgan, Kazakhstan (45.0177° N, 78.3804° E). The indoor SB facility (ISBF) was established in an area of 50 m^2^ (5 X 10 m) and was used for initial experiments (**Figures 1A, B**). Comparatively, the glasshouse SB facility (GSBF) spans 300 m^2^ area and divided into six 40 m^2^ rooms, located side-by-side through the corridor, for easy maintenance of the microclimate inside the different SB glasshouse chambers (**Figures 1C–E**). The ISBF and each room of GSBF was equipped with water supply, LED grow lighting systems and Split Air Conditioner (cooling and heating all in one), for creating optimal conditions for plant growth. The ISBF was fitted with six steel benches (1.3 x 3 m) and each room of GSBF with five (1.5 x 3 m) rolling benches with adjustable height. In both facilities, two LED grow lights (Elixia LX602C Heliospectra, Sweden) were installed vertically on top of each plant growing bench with the distance of 1.0 m. The distance between lights on adjacent benches was 1.7 m. The arrangements ensured uniform light distribution on benchtop. The main heating system and electric switchboard were installed in the 30 m^2^ buffer zone (**Figure 1**).

### 2.2 Development of effective potting mixture and its assessment

The potting mix significantly affects the balanced development of the plant’s root system under SB. To find the best suitable plant growing substrate, we developed 16 different treatments (T0-T15) comprised of various combinations of growing substances such as unfertilized peatmoss (UFPM), factory-fertilized peatmoss (FFPM), compost, sand, soil, grit, zoo humus, bio humus, coconut coir, bark, and soil (**Table 1**). The wheat variety ‘Pamyati Azieva’ was sown in three replicates per treatment and grown in a randomized experimental design to assess the efficacy of 16 newly developed plant-growing substrates. ‘Pamayti Azieva’ is a variety of Russian origin and, registered and commercially grown in Kazakhstan.

**Table 1:** Composition of substrate treatments used in the experiment.

Treatment	Substrate composition (%)
T0	FFPM 75%, perlite 25%
T1	FFPM 66.7%, perlite 16.7%, sand 16.7%
T2	FFPM 66.7%, perlite 16.7%, grit 16.7%
T3	FFPM 60%, perlite 20%, coconut coir 20%
T4	FFPM 60%, perlite 20%, bark 20%
T5	FFPM 60%, perlite 20%, soil 20%
T6	FFPM 60%, perlite 20%, zoo humus 20%
T7	FFPM 60%, perlite 20%, compost 20%
T8	Compost 50%, coconut coir 33.3%, perlite 16.7%
T9	FFPM 29.6%, compost 29.6%, perlite 14.8%, bark 14.8%, bio humus 7.4%, grit 3.7%
T10	UFPM 60%, perlite 20%, bio humus 20%
T11	FFPM 60%, perlite 20%, bio humus 20%
T12	Bio humus 75%, perlite 25%
T13	UFPM 75%, perlite 25%
T14	FFPM 65%, perlite 18%, sand 12%, bio humus 7%
T15	UFPM 75%, perlite 25%

The unfertilized peatmoss, fertilized peatmoss, compost, and bio humus served as the base substances for the treatments. The UFPM and FFPM had a pH of 5.5–6.5, but the FFPM contained additional NPK fertilizers: nitrogen (NH4+ + NO_3_), phosphate (P_2_O_5_), and potassium (K_2_O) at concentrations of 140 mg L^-1^, 160 mg L^-1,^ and 270 mg L^-1^, respectively. Similarly, the pH of the compost was 5.5 to 6.5, with additional nutrients present at concentrations of 100 mg total nitrogen L^-1^, 175 mg phosphorus L^-1^, and 225 mg potassium L^-1^. Bio humus had the same pH, but the nutrient levels were supplied at reduced dosages: 14 mg nitrogen, 16 mg phosphorus, and 24 mg potassium L^-1^. No additional micro- and macronutrients were used at this stage.

### 2.3 Potting mix nutrient management strategies

Once the suitable growing substrate was identified, the soil nutrient management strategy of T14, as the most suitable potting mix, included two steps: i) identification of the importance of NPK (Nitrogen-Phosphorus-Potassium) supplementation, and ii) identification of optimal timing and dosage of nutrients.

- For determining the importance of NPK supplementation, the wheat variety ‘Paragon’ (UK) was sown and grown in a 24-well tray (two seeds per well) using UFPM and FFPM. These substrates were the most suitable base for developing a growing mix, compared with compost and bio humus. Half of the tray (12 wells) was filled with UFPM, and the other half with FFPM. In the end of the experiment, yield components such as plant height (PH), spike length (SL), number of spikelets per main spike (NSMS), main spike weight (MSW), number of kernels per main spike (NKS), grain weight per main spike (GWMS), thousand grain weight (TGW), number of kernels per plant (NKP), grain weight per plant (GWP), seed length (SEL) and seed width (SEW) were analyzed. To evaluate seed architectures (SEM, SEW, TGW), the Marvin (Germany) seed phenotyping platform was used.
- For the identification of optimal NPK dosage and timing, four different time intervals/treatments of NPK application, every 10-day (granules), 14-day (granules), 21-day (granules), and 21-day (liquid), and non-fertilized (NF) control without added NPK supplementation were assessed on seven wheat varieties/lines (‘Paragon’, ‘Aina’, ‘Pamyati Azieva’, ‘Alchemy’ (*Rht-D1*), ‘Saitama’ (*Rht-B1b*), ‘Robigus’ (*Rht-B1b*), ‘H117’ (*RhtD1bxB1b*)). Each wheat cultivar was sown in 1L plastic pots in three replications and grown in complete randomized blocks (CRB) under GSBF conditions. Plants were supplied with fertilizers as sources of nitrogen, phosphorus and potassium. Specifically, urea ((CO(NH_2_)_2_) was applied as the primary source of nitrogen (N), ammophos (monoammonium phosphate, MAP) containing 52% P_2_O_5_ and 12% N (FERTIKA) was used as phosphorus (P) source and potassium nitrate KNO_3_, containing 13.5% N and 45.8% K_2_O, was applied as the source of potassium (K). Fertilizers were supplied at aforementioned 4 different time intervals (10d, 14d, 21d, 21d_L) at rates of 0.1g ((CO(NH_2_)_2_), 0.3g (P_2_O_5_), and 0.6g (K_2_O) per L^-1^. For the 21d_L treatment, this amount of nutrients was weighed and dissolved in potable municipal water that was used for plant watering. The irrigation was continued until early maturity to obtain fully developed, healthy seeds. At maturity, yield components such as spike length (SL), number of spikelets per main spike (NSMS), effective tiller number (ETN), number of kernels per plant (NKP), grain weight per plant (GWP), thousand grain weight (TGW), seed width (SEW), and seed length (SEL) were assessed.

### 2.4 Light-benchtop distance experiment

**Figure 2:**
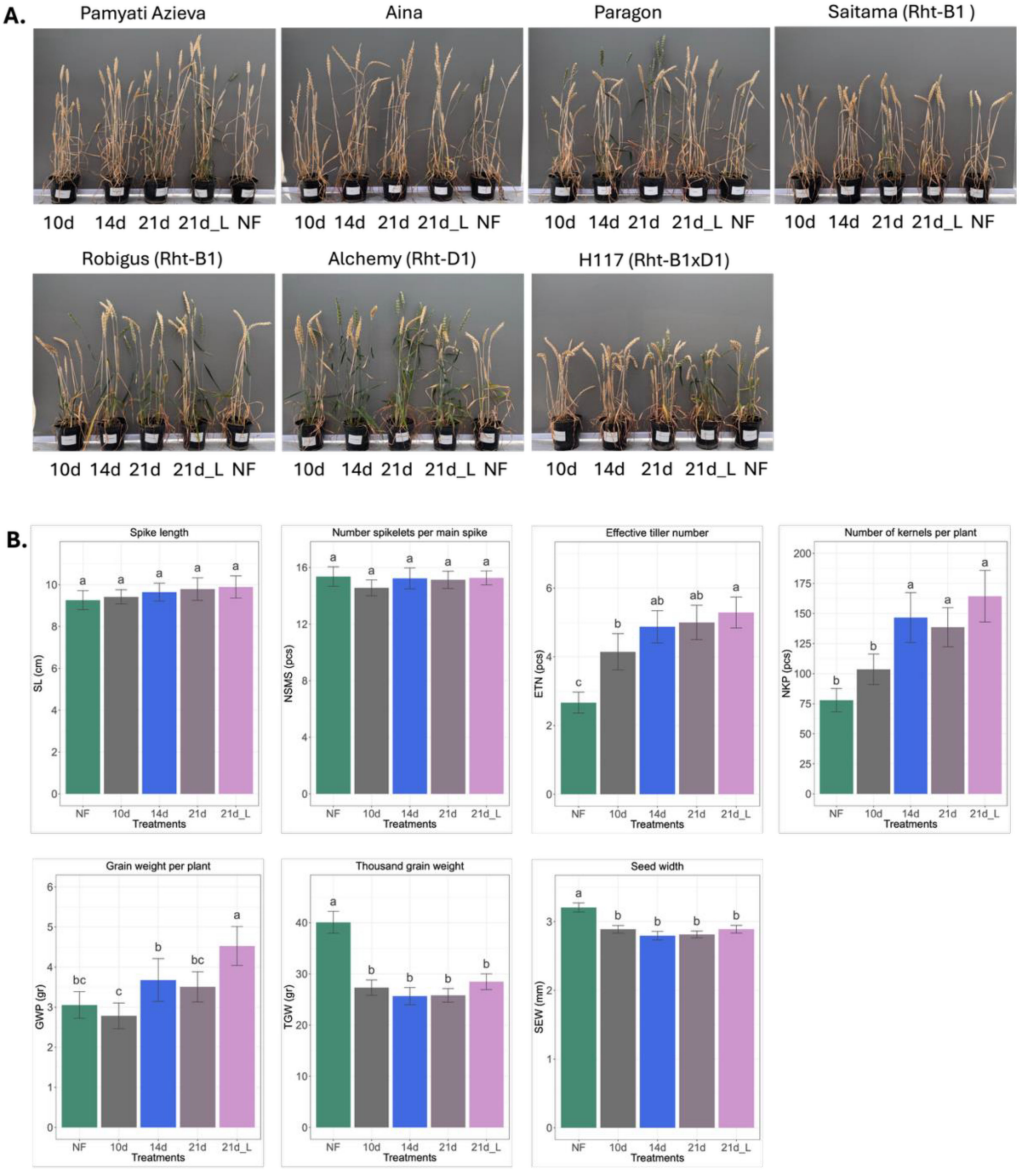
Soil nutrient management. **(A)** Wheat genotypes ‘Pamyati Azieva’, ‘Aina’, ‘Paragon’, ‘Saitama’ (Rht-B1b), ‘Robigus’ (Rht-B1b), ‘Alchemy’ (Rht-D1b) and ‘H117’ (RhtD1bxB1b) grown under five different NPK application treatments. **(B)** Evaluated yield components. The letters on top of bars demonstrate significant differences between treatments as determined by Tukey’s HSD test at p < 0.05. Summary statistics show ± standard error of the mean (SEM).

Light-bench distance is a critical criterion when plants are subjected to SB (Watson et al., 2018). Therefore, we have grown three wheat varieties ‘Paragon’ (UK), ‘Pirsbak-21’ (PAK), and ‘CN X Borlaug’ (PAK) under two, 1.5 and 2.0-meter, light-bench distances. These distances were chosen to generate variation in the light intensity received by wheat plants and to evaluate growth and developmental dynamics under indoor SB conditions. The genotypes were sown in 2 L pots in two replicates and grown in a randomized experiment under ISBF (**Figure 2**). Plants were supplemented with a foliar spray of calcium nitrate (Ca(NO_3_)_2_) and copper (Cu) with the concentrations of 1gr/L^-1^ and 100mL/L^-1^ respectively and were hand-watered once a day (evening), without causing waterlogging issues, providing enough aeration for normal root development, which was stopped at the grain filling stage to accelerate the ripening. Following the phenotyping protocol used in previous experiments, the main yield indicators, including TGW, ETN, NKP, SEL, and SEW, were assessed. The same experiment was conducted in GSBF, but we did not observe a significant variation between the two light-bench treatments.

### 2.5 Light spectrum, light intensity, cooling, and heating setups

The LED grow lights (Elixia LX602C Heliospectra, Sweden) provided adjustable spectral channels including blue (450 nm), red (660 nm), far red (735nm), and white (5700 K). The intensity of each spectral channel was initially set to 500 from germination to early tillering, then gradually increased to 600 during late tillering and 700 during the early booting stages, allowing precise regulation of light quality. The power consumption ranged from 10.5 to 600 W, depending on the selected light intensity and channel settings. The temperature inside the facility was kept at +22 °C (+/- 1-1.5) for 22 h and at +18 °C (+/- 1-1.5) for 2 h during the day and night, respectively. Air circulation was supported by opening the side/top windows or/and/or doors of the facility (**Figure 1E**).

### 2.6 Early harvest and seed drying

A germination test was conducted on four wheat grains harvested at different developmental phases – 15, 20, 25, 30, and 35 days after flowering or at the early grain filling stage. Early harvested seeds were oven-dried at +35 °C for three days. Then the seeds were sown in Petri dishes, soaked in distilled water, and refrigerated in dark at +4 °C for 2 days to induce cold shock. Afterward, the seeds were transferred to the climate chamber (Being Technology Co., Ltd., China) to germinate at +22 °C with 50% humidity and a 12-hour day-night cycle for three days prior to being sown and grown in potting soil.

### 2.7 Data analysis and figure generation

**Figure 3:**
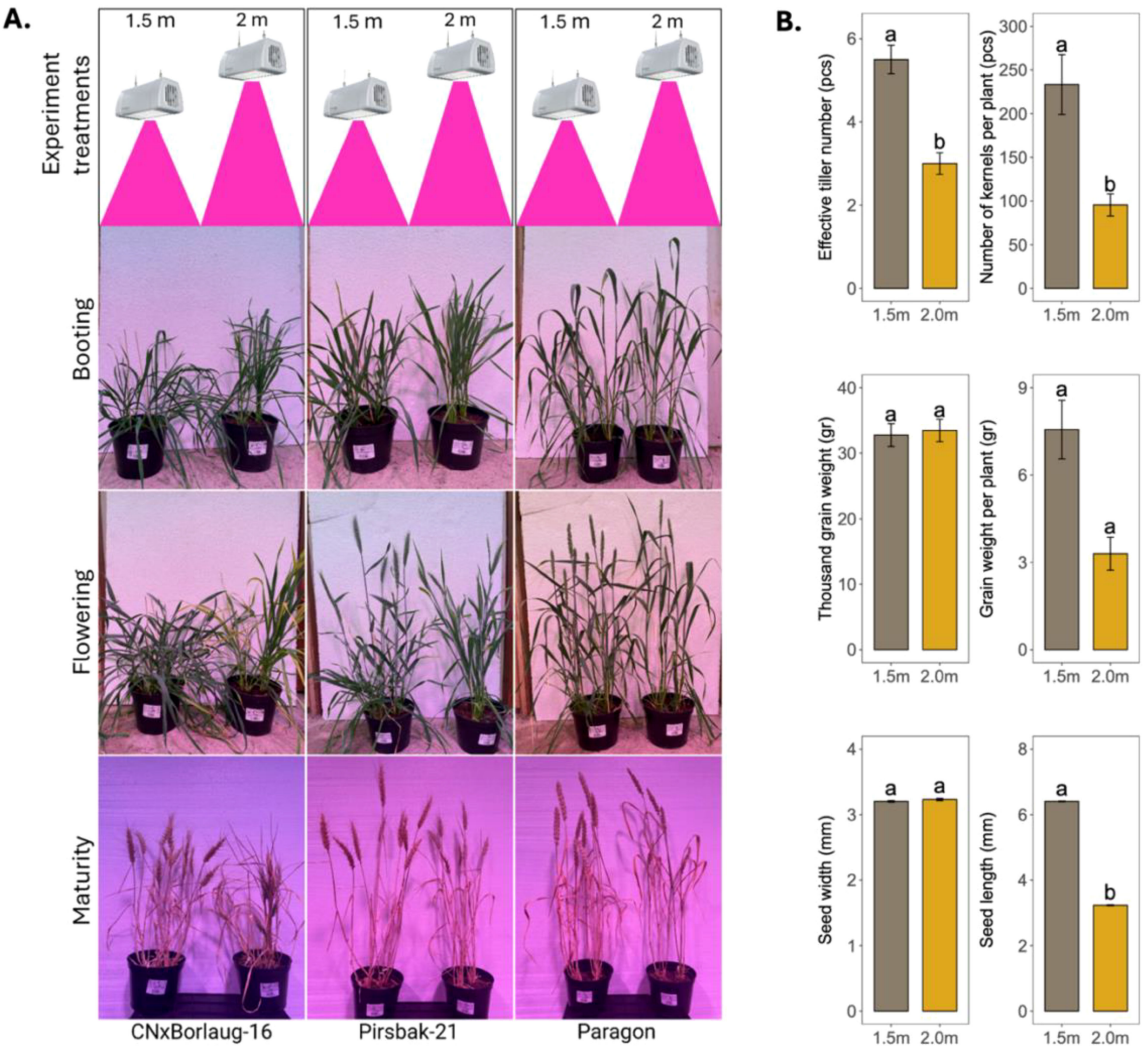
**(A)** Light effects on development and **(B)** yield components. The letters on top of bars indicate statistical significance level at p<0.05. Summary statistics show ± standard error of the mean (SEM).

**Figure 4:**
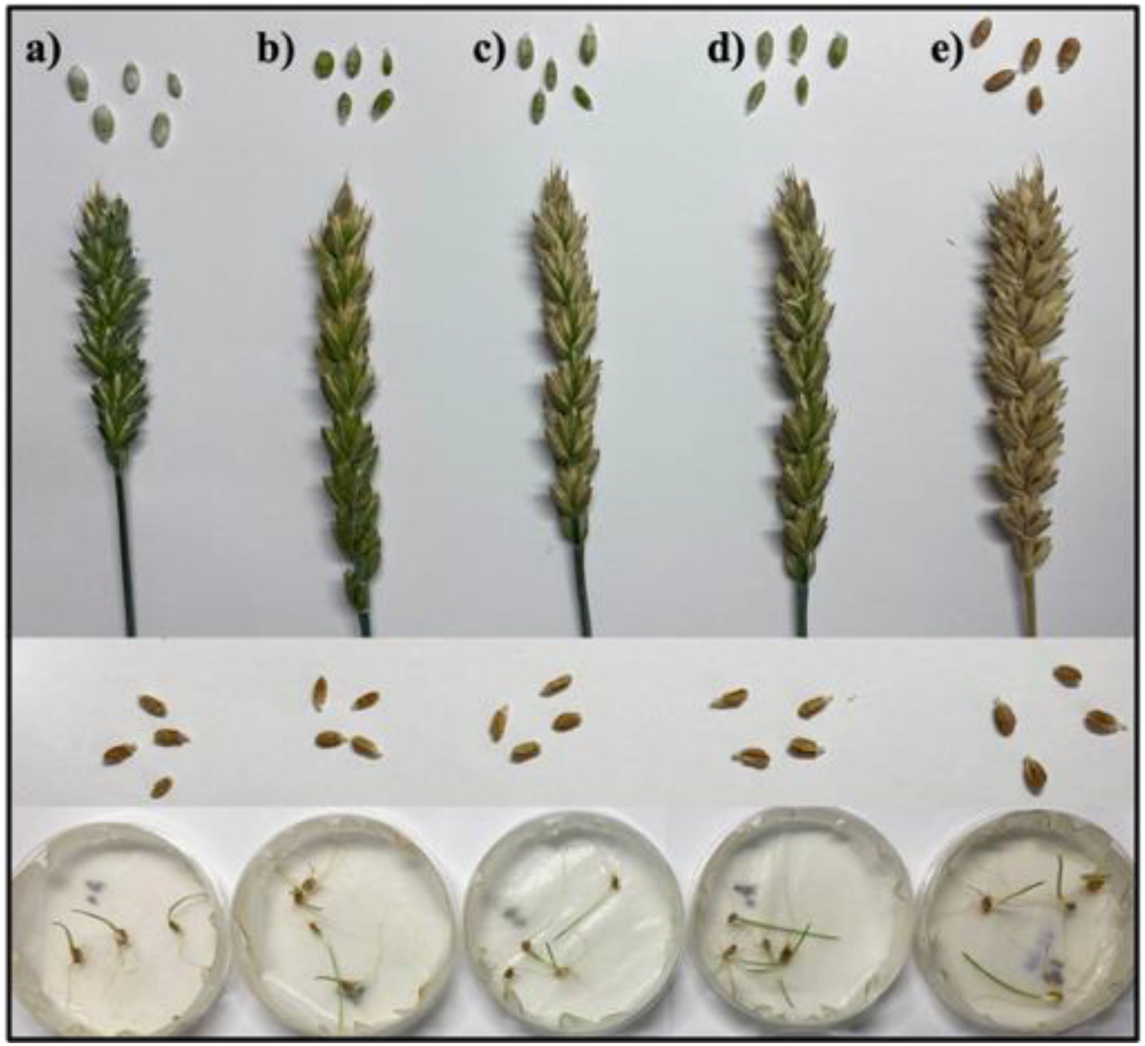
Germination test of early harvested wheat seeds: **(A–E)** represent the seeds harvested 15, 20, 25, 30 and 35 days after flowering respectively under speed breeding conditions.

The results obtained from the NPK supplementation and light-bench distance experiments were analyzed using a two-sample t-test. A one-way ANOVA model was fitted to the results of the soil nutrient management experiment. The ANOVA model was then subjected to a multiple comparisons test using Tukey’s Honestly Significant Difference (HSD) for determining the significantly different group means. The assumption of homogeneity of variances was tested using Bartlett’s test. Statistical analyses and the plot generation were conducted in R using the *ggplot* package. **Figures 1E**, **3A**, **4** were created in BioRender (https://BioRender.com).

## 3 Results

### 3.1 Comparative performance of potting substrate quality characteristics

**Figure 5:**
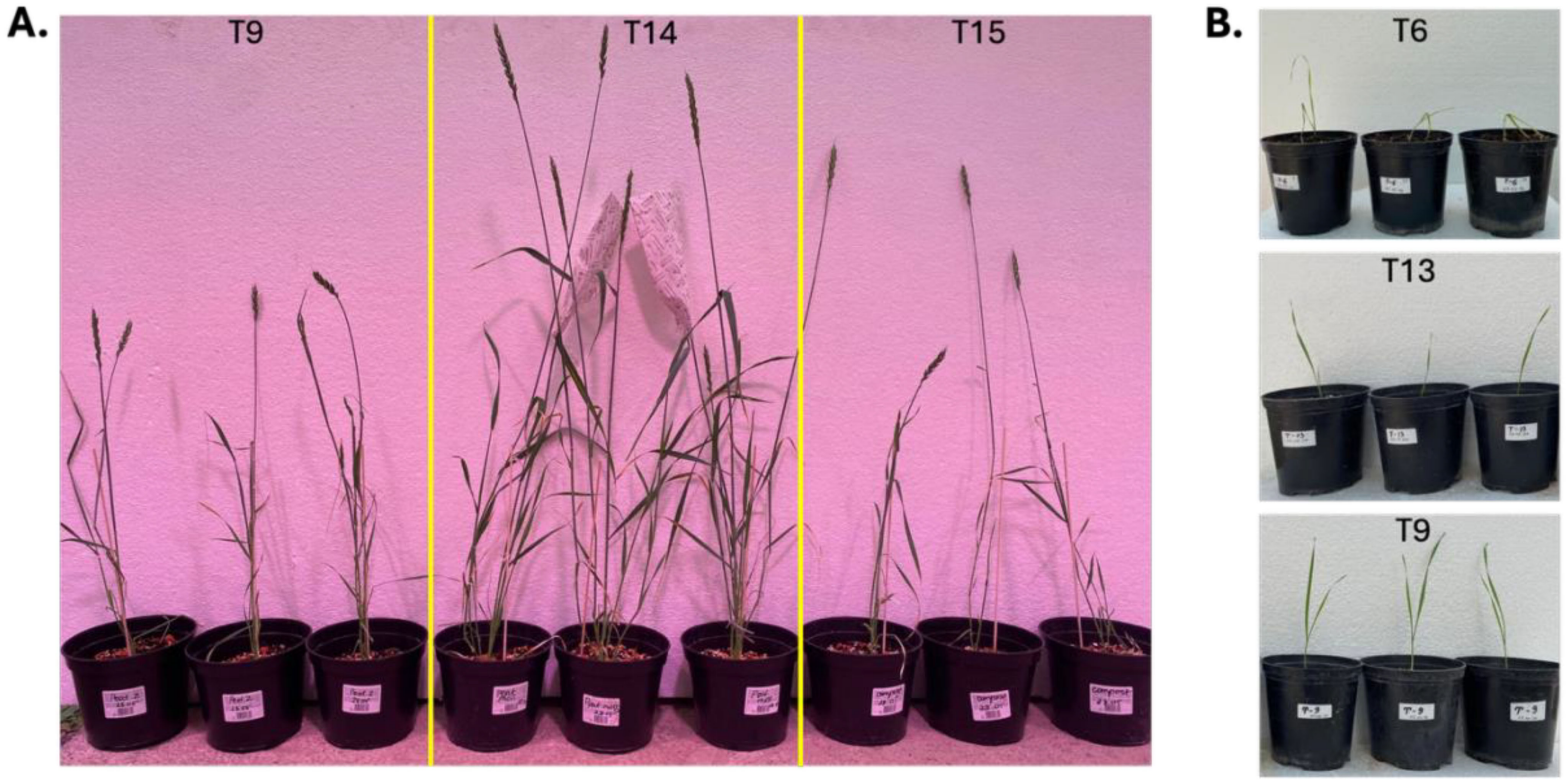
Soil treatments. **(A)** From left to right: T9, T14 and T15, and **(B)** T13, T9 and T6 are shown selectively for the sake of comparison.

The high-quality potting mixture is of vital importance for maximizing the plant survival, growth and productivity under stressed SB conditions. Among developed sixteen different potting substrates to grow wheat plants under SB, three – T9 (composed of 29.6% FFPM, 29.6% compost, 14.8% perlite, 14.8% bark, 7.4% bio humus and 3.7% grit), T14 (containing 65% FFPM, 18% perlite, 12% sand, and 7% bio humus), and T15 (with 75% UFPM and 25% perlite) – were favorable for normal plant growth and development, but all three exhibited a poor plant tillering at this stage of protocol optimization (**Figure 5A**). Plants grown in T14, compared with those in T9 and T15, pronounced better physiological performance across developmental stages. Specifically, T14 was associated with balanced water-holding and aeration capacities, which are essential in SB systems, where accelerated growth is required. The improvement in plant tillering in T14 was further enhanced through nutrient supplementation in the “Soil Nutrient Management Strategies” experiment.

The remaining thirteen combinations demonstrated frequently encountered soil-related constraints such as waterlogging and limited aeration, which eventually led to mold and algal growth. Wheat cultivars grown under these treatments, although they germinated well, exhibited poor establishment and severe stress signs throughout the development. Most plants did not survive and perished in different growth stages in these treatments (**Figure 5B**).

### 3.2 Growth responses to different nutrient application rates and schedules

**Figure 6:**
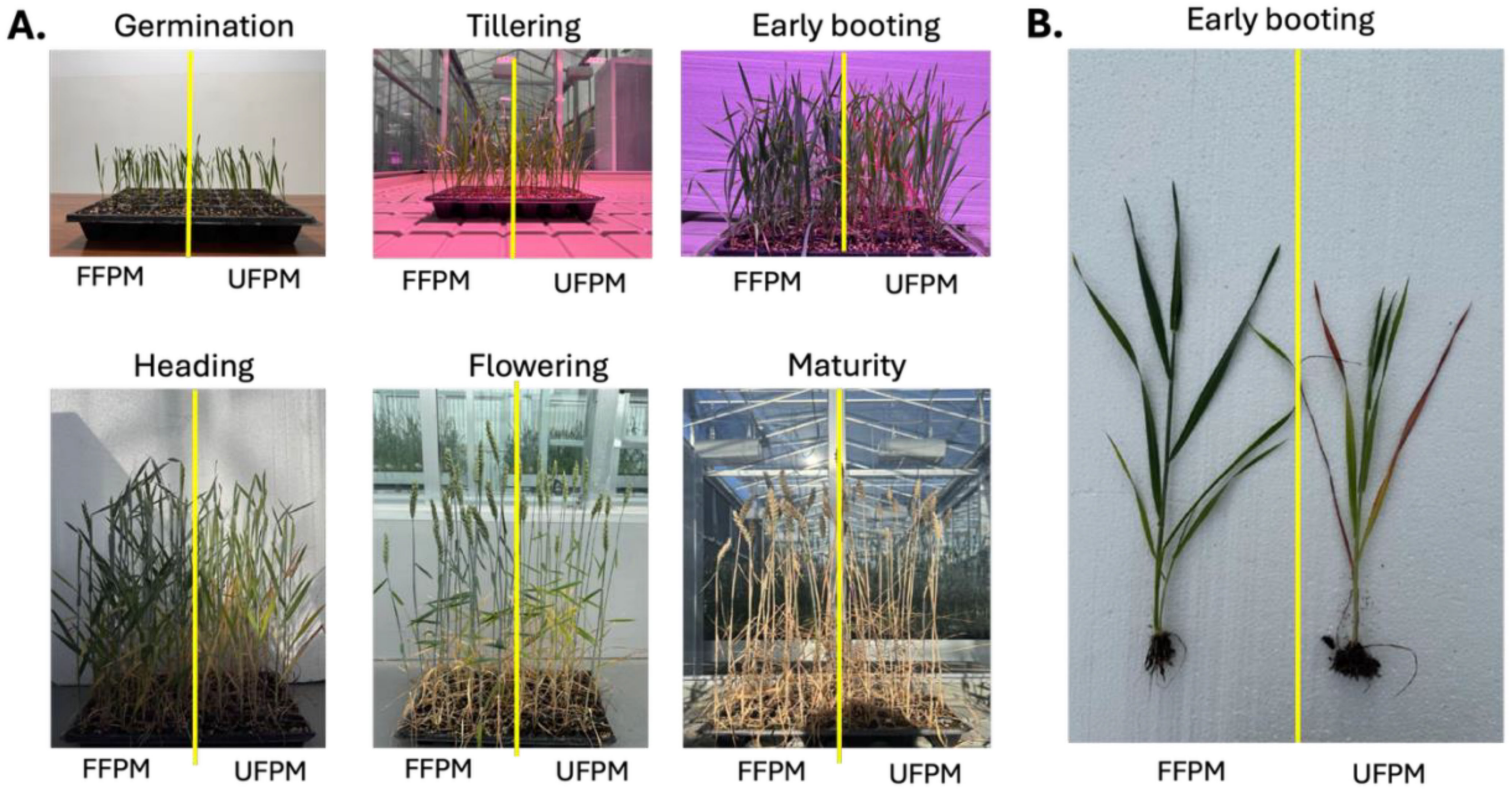
**(A)** Wheat plants grown in factory-fertilized peatmoss (FFPM) and unfertilized peatmoss (UFPM). Cultivar ‘Paragon’. **(B)** Plants grown in UFPM showed phosphorus deficiency compared to plants grown FFPM after late tillering stage.

**Figure 7:**
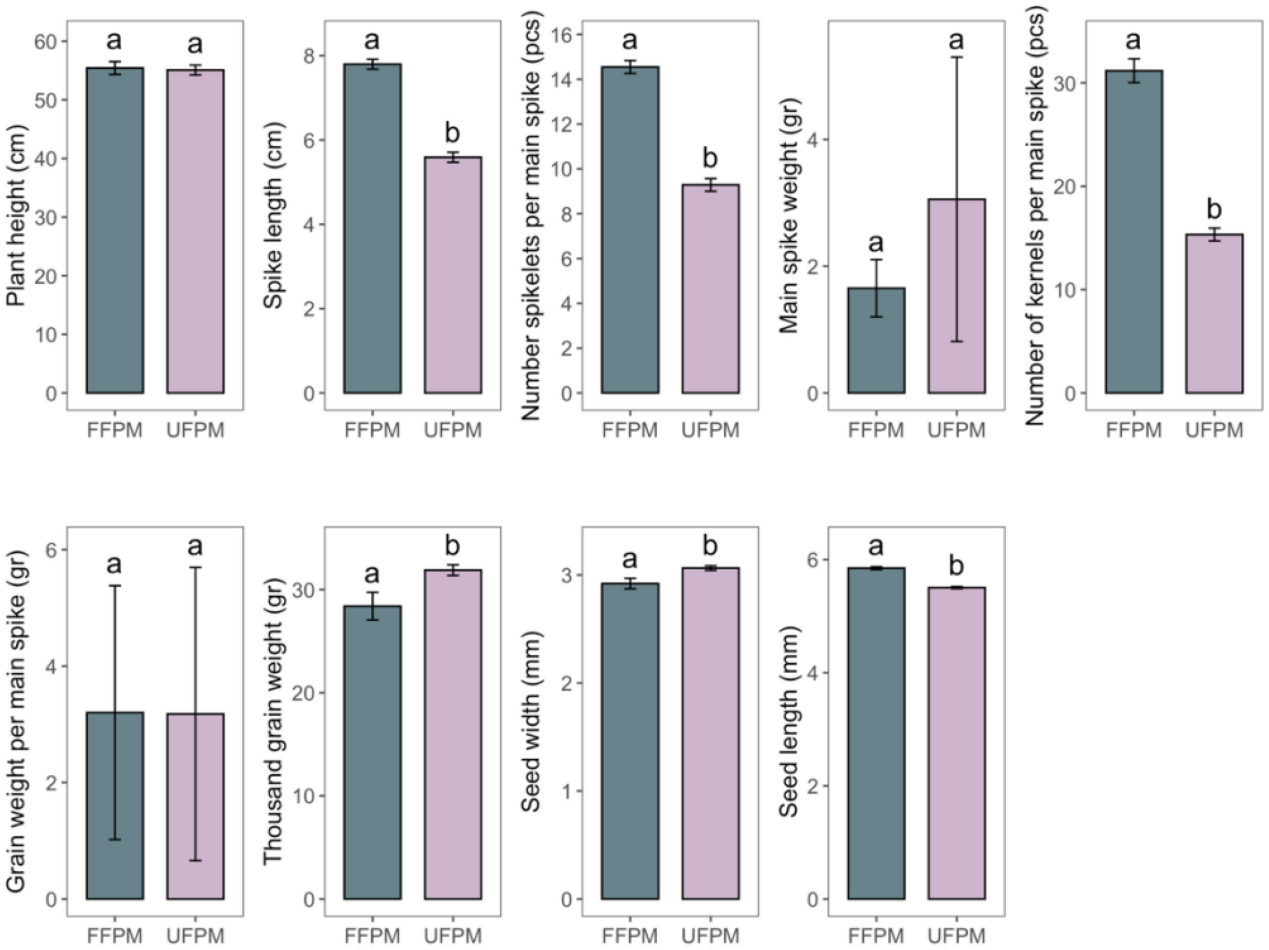
The effect of NPK supplementation under speed breeding. The letters on top of bars indicate statistical significance level at p<0.05. Summary statistics show ± standard error of the mean (SEM).

- Determination of the importance of NPK supplementation: During germination and tillering phases, no phenotypic difference was noticed between wheat plants grown in FFPM and UFPM (**Figure 6A**). They demonstrated normal physiological development. However, after the late tillering stage, plants grown in UFPM showed a phosphorus (P_2_O_5_) deficiency, while plants on the other half of the tray with FFPM exhibited normal physiological performance (**Figures 6A, B**). The same symptom of P_2_O_5_ deficiency appeared on plants grown in FFPM after 5–7 days. The application of monoammonium phosphate (52% P_2_O_5_ and 12% N) when phosphorus deficiency was identified restored normal physiological growth in plants grown in FFPM. This MAP supplementation of FFPM-grown plants was continued until the ripening stage, at which point N and P_2_O_5_ deficiencies became apparent. In comparison, UFPM-grown plants, the other half of the tray, were kept under nutrient deficiency until maturity to explore how nutrient deficiency would affect plant development and final grain yield under SB. After ripening, we harvested FFPM- and UFPM-grown plants and collected primary data for comparison. Data analysis showed that plants grown under fertilized conditions had significantly longer ears and seeds, and higher spikelet numbers and kernel numbers per main spike compared to plants grown without extra nutrients. Although additional fertilizer supplementation improved grain number and other yield components, it negatively affected SWE and TGW – the main yield indicator. Moreover, plant height and grain weight were nearly identical between the two treatments, indicating the need for further optimization of nutrient dosage and application timing (**Figure 7**).
- Identification of the optimal timing and dosage of nutrients: Under SB conditions, additional NPK supplementation significantly increased grain yield and its main components. For example, experimental results clearly demonstrated that applying nutrients in liquid form at 21-day intervals (21d_L) significantly improved ETN by 98.4% (p = 1.3x10^-5^), NKP by 110% (p = 1x10^-8^), and, importantly, GWP by 48.2% (p = 2x10^-5^) compared with the non-fertilized control (NF). Likewise, significant improvements were observed with 14d and 21d treatments, which increased ETN by 82.7% (p = 1x10^-8^) and 87.5% (p = 1x10^-8^) respectively, and NKP by 88.2% (p < 6x10^-8^) and 77,9% (p < 1x10^-6^) respectively. However, in contrast to 21d_L, the mean differences in GWP for the 14d (p = 2x10^-1^) and 21d (p = 6x10^-1^) treatments were not statistically significant compared with NF. Frequent fertilizer application at 10-day intervals (10d) adversely affected yield components. No differences were observed among treatment means for SL, NSMS, and SEL. Comparatively, unfertilized plants set plump (wider) seeds than fertilized plants across all treatments. Therefore, NPK supplementation had a significant negative effect on TGW and SEW, but without an adverse impact on overall grain yield (**Figures 2A, B**).

Overall, the NPK application was administered six, four, and three times for the 10d, 14d, and 21d/21d_L treatments, respectively. In the 10d treatment, six NPK application events occurred at six days after germination; four, fourteen, and twenty-four days after tillering; and three and thirteen days after flowering. In the 14-day treatment, four NPK application timings were applied at ten days after germination, twelve days after tillering, and twenty-six days after heading. In contrast, three NPK application intervals of the 21d and 21d_L treatments occurred at five and twenty-six days after tillering, and seventeen days after flowering.

### 3.3 Optimal light–bench distance

Optimal light–bench distance created favorable conditions for balanced plant growth under both ISBF and GSBF conditions. Three wheat genotypes evaluated under two light-bench distances (1.5 and 2.0 m) exhibited very similar growth dynamics from germination to tillering. However, a clear difference in plant stature was observed between the two light-bench distance treatments at stem elongation and early booting. From heading onward, wheat genotypes treated under the 2.0 m light-bench condition showed a significant growth retardation (**Figure 3A**).

When the effect of two light treatments on yield components was compared, the tillering capacity of plants grown under the 1.5 m treatment was significantly higher than that of the 2.0 m condition. This resulted in an 83.3% increase in ETN (p = 2x10^-4^), a 145% in NKP (p = 8x10^-3^) and a 127% in GWP (p = 6x10^-3^) without compromising thousand grain weight (TGW, p = 8x10^-1^), highlighting a potential positive effect of proximity to a 1.5 m light-bench under SB conditions (**Figure 3B**).

### 3.4 Influence of light quality, light intensity, and temperature setups on plant development

**Figure 8:**
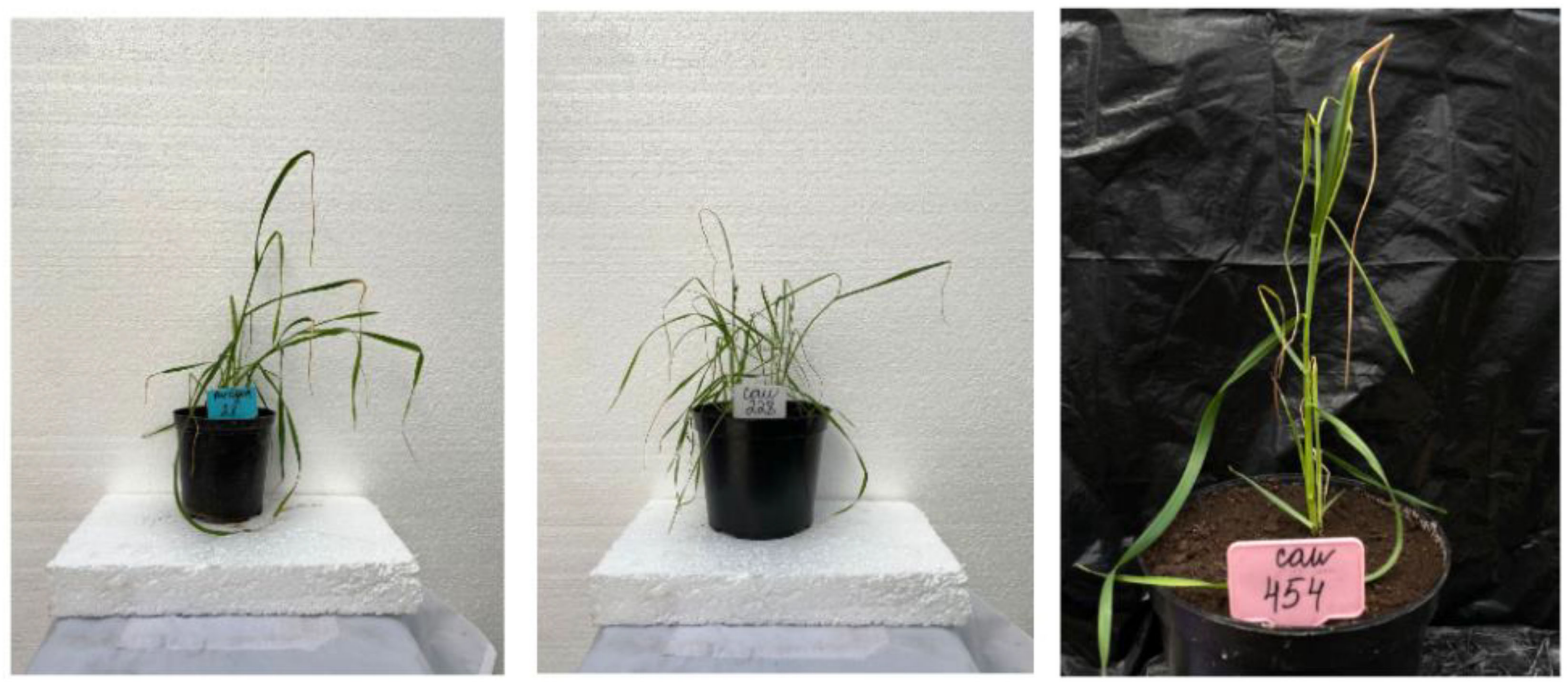
Temperature stress on wheat plants observed under SB.

Light is a key component of speed breeding, and its intensity influences plant growth, development, and productivity. During protocol optimization, we observed that light intensities in the range of 500–700, depending on the developmental stage, supported healthy wheat growth under SB conditions (**Figure 8**).

**Figure 9:**
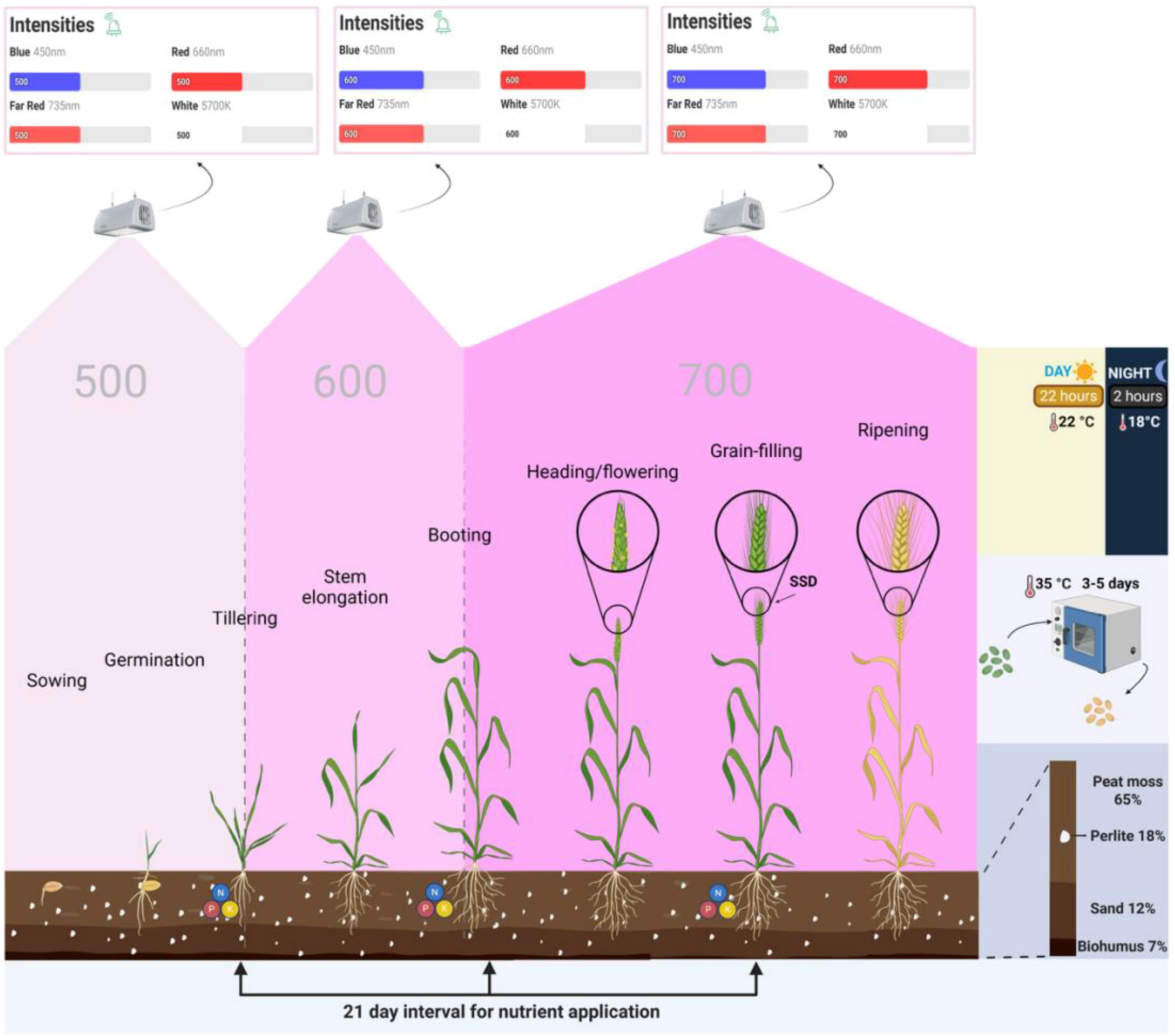
Optimized SB protocol: light intensity, light spectrum, temperature setups, seed drying conditions, potting substrate and NPK application schedule.

Like light, temperature control is equally important for speed growth. We found that it is acceptable when the temperature fluctuates at around 1-1.5 °C from the ideal condition (22 °C) during the daytime. However, it was difficult to maintain optimal thermal conditions in GSBF when outdoor temperatures were highest (above 35 °C) during summertime. We observed that the short-term, slight increase in temperature (24-25 °C up to two hours per day) in SB did not result in significant adverse effects on overall plant health and physiology. However, when the temperature increase was prolonged, plants exhibited severe signs of stress, eventually leading to plant death (**Figure 9**). Lower temperatures (15-17 °C) for two hours at night did not negatively affect plant growth but slightly delayed plant development.

### 3.5 Germination of early harvested wheat seeds

Achieving rapid generation advancement under speed breeding is crucial. Germination test results for wheat seeds harvested at different time points, 15, 20, 25, 30, 35 days after flowering, showed 100% viability. Seeds developed root and shoot systems without any physiological abnormalities (**Figure 4**). Likewise, the germinated seeds grew well when transferred to pots containing T14 (data are not shown, as plants were destroyed after tillering). Accordingly, our in-house-developed SB protocol, which integrates optimized light intensity, spectral composition, light-bench distance, potting substrate, and NPK application schedule, was well-suited for the cultivation of wheat under SB conditions. An overview of the SB protocol is provided in **Figure 8**.

## 4 Discussion

### 4.1 Importance of high-quality potting substrate under speed breeding conditions

Balanced composition of potting substrate enhances its aeration, water infiltration, water retention, and nutrient uptake capacities, thereby positively influencing plant growth and development under controlled environments, including SB (Verhagen, 2013; Bayabil et al., 2021; Bayhan et al., 2025). The SB potting mix used by research teams from the UK (John Innes Centre) and Australia (University of Queensland) was carefully designed and supplied by Petersfield Growing (Leicester, UK) and Central Glasshouse Services of University of Queensland (Australia) respectively (Ghosh et al., 2018; Watson et al., 2018). The potting mix supplied by Petersfield Growing contained peat with a specific size, sterilized soil, and horticultural grit in addition to fertilizers and insecticides. The availability of these potting substrate components in developing or underdeveloped nations, including those in Central Asia, appears to be limited. If available, they could be very costly. In comparison, the sowing mix designed by Central Glasshouse Services of the University of Queensland consisted of composted pine bark (0-5mm) and coco peat, both of which are sold in small amounts and are very expensive. Treatments containing coconut coir and bark were not effective for wheat in this study, perhaps due to allelopathic effects (Khan et al., 2023). Most of the potting substrates developed in this study either failed to support rapid plant development or were unsuitable for growing wheat under SB conditions, primarily due to waterlogging (**Figure 5B**). It is well known that waterlogging reduces O_2_ transfer, thereby decreasing nitrogen availability and negatively impacting overall plant growth, development, and productivity, including in wheat (Nguyen et al., 2018; Manghwar et al., 2024). When potting soil becomes excessively compact and heavy, it leads to weak root development (Jin et al., 2023). The most effective potting substrate combination we developed contained 65% FFPM, 18% perlite, 12% sand, and 7% bio humus L^-1^. It demonstrated improved water-holding and aeration capacity, ultimately supporting healthy root development compared to other potting substrate treatments (**Figures 5A, B**). Although the developed substrate reduced costs by 64.1% and 29.3% relative to UK and Australian SB protocols, respectively, its cost-effectiveness may vary in other parts of the world.

### 4.2 Optimal timing and dosage of NPK supplementation under speed breeding conditions

Due to accelerated vegetative growth, physiological development, and stress conditions under SB, plants require additional macro and micronutrients to complete their life cycle. A deficiency of these essential elements may adversely affect and slow overall plant development in SB (Watson et al., 2018; Blinkov et al., 2025). Our soil nutrient management strategies of potting substrate that showed the highest performance (T14) among treatment included two steps: i) identification of the importance of NPK supplementation of FFPM serving as a base component for T14, and ii) identification of optimal timing and dosage of nutrients. In the first half of the experiment, we found that the minimal nutrient content of FFPM was insufficient to support healthy wheat plant growth throughout development (**Figures 6A, B**). In the second half, several treatments that were designed for the identification of timing and dosage of NPK supplementation under SB highlighted that nutrients applied at rates of 0.1g ((CO(NH_2_)_2_), 0.3g (P_2_O_5_), and 0.6g (K_2_O) per L^-1^ at 21-day intervals significantly improved the quality of high-performing T14 potting substrate and yield components of wheat plants grown in it (**Figures 2A, B**).

While plants grown under all NPK-supplied treatments had lower TGW than NF, there was a significant increase in ETN, NKP, and, most importantly, GWP. It also promoted overall biomass. This highlights the importance of additional NPK supplementation of FFPM, the main component of T14 (65%), for which we did not observe a significant difference in GWP in the experiment compared with UFPM. These findings are in line with previous experimental results on the importance of NPK supplementation of nutrient-minimal potting substrates and on NPK-driven biomass accumulation under controlled conditions (Epie and Maral, 2018; Yang et al., 2021). In the 21d_L treatment, the first NPK application coincided with the tillering stage. The tillering stage is a critical phase in determining the plant’s yield potential because it is associated with the formation of lateral shoots and root development (Takeno, 2016). The second and third NPK treatments overlapped with the flowering and grain filling stages at 42 and 63 days, respectively (**Figure 8**). The application of nitrogen, phosphorus, and potassium at these critical stages of plant development improves grain weight and quality by extending the grain-filling stage and promoting assimilate remobilization (Li et al., 2025; Yang et al., 2025; Liu et al., 2026). The excessive application of nutrients (NPK) in the 10d treatment resulted in a significant reduction in grain weight and yield components compared to other treatments (**Figure 2B**). This observation strongly supports earlier reports that when NPK above the optimum threshold, grain yield is reduced and nitrate leaching increases (Wang et al., 2011; Yang et al., 2025). We observed 48.2% and 110% increase in GWP (p = 2x10-5) and NKP (p = 1x10^-8^) between NPK supplemented potting mix and the control potting mix (NF). However, additional fertilized application had negative impact on TGW due to a negative relationship that could arise from competition among growing grains for limited assimilates during grain filling and/or the trade-off between grain weight and grain number (Slafer et al., 2022; Vicentin et al., 2024).

Although sufficient watering throughout the developmental stage, from sowing to harvesting, allowed us to observe the maximum effects of different NPK treatments on grain yield and to conduct comparative analysis to identify differences between them, it delayed physiological maturity under SB conditions. Nevertheless, the flowering time was observed at day 42, which is specific for SB (Ghosh et al., 2018; Watson et al., 2018). The seeds collected on the 15^th^ day after flowering were applicable for sowing and were viable (**Figure 4**). As nitrogen use efficiency drops significantly under low moisture conditions (Epie and Maral, 2018). Our watering strategy enabled a reliable comparison of variations among the NKP treatments. However, in rapid generation advancement and light-benchtop distance experiments, the seed-to-seed duration ranged from 58 to 63 days, depending on the genotype, with heading time scored at days 28 and 32 from sowing (**Figure 3**).

### 4.3 Optimal light parameters

In addition to well-controlled temperature, maintaining optimal light installation, that is, light–light distance, light–bench distance, light spectrum, and its intensity collectively create favorable conditions for balanced plant growth under both indoor and greenhouse SB conditions. The previous reports highlighted two light-bench distances, ~1.5 m and ~2.5 m by AUS and UK research teams, respectively, as optimal (Ghosh et al., 2018; Watson et al., 2018). In this study, we found that ~1.5m light-bench spacing seems to be ideal for wheat cultivation under both indoor and greenhouse SB facilities in Central Asia, Kazakhstan. Although light-bench spacing had no effect on physiological development until tillering under SB conditions, wheat plants grown far from the light source showed significant growth retardation when they entered the stem elongation stage (**Figures 3A, B**). The absence of physiological differences between light-bench distance treatments (~1.5 m and ~2.5 m) at pre-tillering is consistent with studies in wheat physiology, indicating limited sensitivity of early vegetative development to light availability (Fischer, 1985; González et al., 2003, 2005). However, stem elongation represents a phase of increased carbon demand, during which reduced radiation availability strongly constrains structural growth, leading to growth retardation (Fischer, 1985).

**Figure 10:**
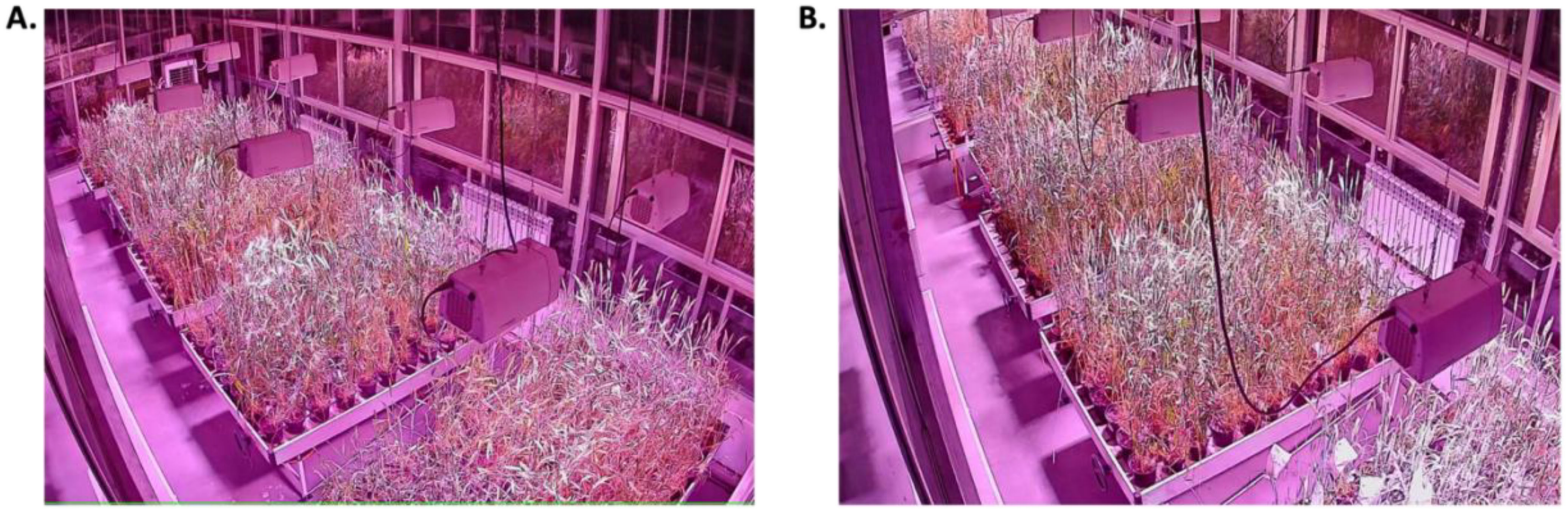
Central Asian wheat panel grown under speed breeding conditions: **(A)** wheat genotypes with spring habit and **(B)** winter and facultative cultivars.

The LED grow lights were installed with spacings of ~1.6 m vertically and ~1.5 m horizontally at the John Innes Centre (UK), compared to ~1.6 m and ~1.0 m at the University of Queensland (AUS) (Ghosh et al., 2018). In our case, the in-between light distances were spaced at ~1.0 m vertically and ~1.7 m horizontally on top of rolling benches measuring 1.5x3 m and adjustable in height. These installation arrangements were adequate to generate even variation in light intensity under both indoor and greenhouse SB conditions. Changes in light intensities (from 500 to 700) across certain developmental phases were suitable for growing healthy wheat plants and for avoiding early signs of stress (leaf tip burning) under SB conditions. In addition, our improved in-house SB protocol, initially fine-tuned on Kazakhstani, Pakistani, and UK wheat varieties, was shown to be effective across broader wheat germplasm panels representing Uzbekistan, Kyrgyzstan, Turkmenistan, Afghanistan, Tajikistan, Russia, Turkiye, Ukraine, and China (**Figure 10**). Nevertheless, external validation is still required. This highlights opportunities to extend the knowledge gained in this work to other developing nations, strengthening their local pre-breeding and breeding processes by adopting the SB technique and supporting sustainable agriculture.

## 5 Conclusions

In this study, we report the establishment of the first SB facility in Kazakhstan - a historically significant place for wheat diversity, breeding and production in Central Asia. As part of the development of an improved speed breeding protocol that is accessible to plant research institutions with limited infrastructure, this study optimized the light-to-benchtop distance and demonstrated the importance of adjusting light spectrum and intensity during the early stages of wheat growth. In addition, a cost-effective potting substrate was developed, and its nutrient composition was optimized. Optimum timing and dosage of NPK supplementation was also identified under speed breeding conditions. As a result, a significant increase was observed in key wheat yield components without compromising rapid generation advancement which is required by SB. The improved protocol proved effective across a diverse wheat germplasm panel, although further validation under independent conditions is still required. Overall, outcomes of this study highlight the importance of using speed breeding technique to develop new improved wheat varieties for Central Asia that can withstand climate change.

## Data Availability

The original contributions presented in the study are included in the article/supplementary material. Further inquiries can be directed to the corresponding authors.
